# Utilizing medical thoracoscopy for the diagnosis of myelomatous pleural effusion: 2 case reports

**DOI:** 10.1002/rcr2.1333

**Published:** 2024-03-25

**Authors:** Khai Lip Ng, Sin Nee Tan, Nai‐Chien Huan, Mona Zaria Nasaruddin, Noriah Othman, Jamalul Azizi Abdul Rahaman

**Affiliations:** ^1^ Department of Medicine Melaka Hospital Melaka Malaysia; ^2^ Department of Pulmonology Serdang Hospital Kajang Malaysia; ^3^ Department of Respiratory Medicine Queen Elizabeth Hospital Kota Kinabalu Malaysia; ^4^ Department of Pathology Serdang Hospital Kajang Malaysia

**Keywords:** medical thoracoscopy, multiple myeloma, myelomatous effusion, pleural effusion

## Abstract

Multiple myeloma (MM) is characterized by neoplastic proliferation of monoclonal antibody producing plasma cells. In clinical practice, pleural effusion is seen in up to 6% of MM patients, with many causative factors. Nevertheless, true myelomatous pleural effusion, defined as infiltration of the pleura by myeloma cells, is very rare. In this case report, we present two patients with biopsy proven myelomatous pleural effusion. The first patient developed myelomatous pleural effusion as initial presentation while the second patient's pleural effusion occurred during disease relapse. In both cases, prompt diagnosis via medical thoracoscopy (MT) followed by early commencement of myeloma specific chemotherapy led to clinical, biochemical, and radiological resolution and therefore were crucial steps in the management of myelomatous pleural effusion.

## Graphical abstract

Myelomatous pleural effusion, defined as infiltration of the pleura by myeloma cells, is very rare. We present two patients with biopsy proven myelomatous pleural effusion diagnosed via medical thoracoscopy (MT) followed by early commencement of myeloma specific chemotherapy which led to clinical, biochemical, and radiological resolution of the disease.

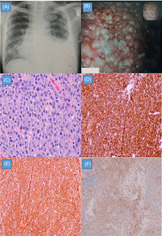

## INTRODUCTION

Multiple myeloma (MM) is characterized by neoplastic proliferation of monoclonal antibody producing plasma cells. MM accounts for 1% of all cancers and about 10% of all malignant haematological diseases and mainly affects the bone marrow although extramedullary sites can be infiltrated as well.^1^ Pleural effusion is seen in up to 6% of patients with MM,^2^ with many potential underlying causes, including renal failure, cardiac failure, pulmonary embolism, amyloidosis, hypoalbuminaemia, pneumonia with parapneumonic effusion and chylothorax due to myelomatous lymphatic obstruction.^2^, ^3^ A true myelomatous pleural effusion, defined as direct infiltration of the pleura by myeloma cells, is rare.^1^, ^4^ Herein, we report two patients with biopsy proven myelomatous pleural effusions, one as initial presentation of MM and the other who developed effusion during disease relapse.

## CASE REPORT

### Case 1

**FIGURE 1:**
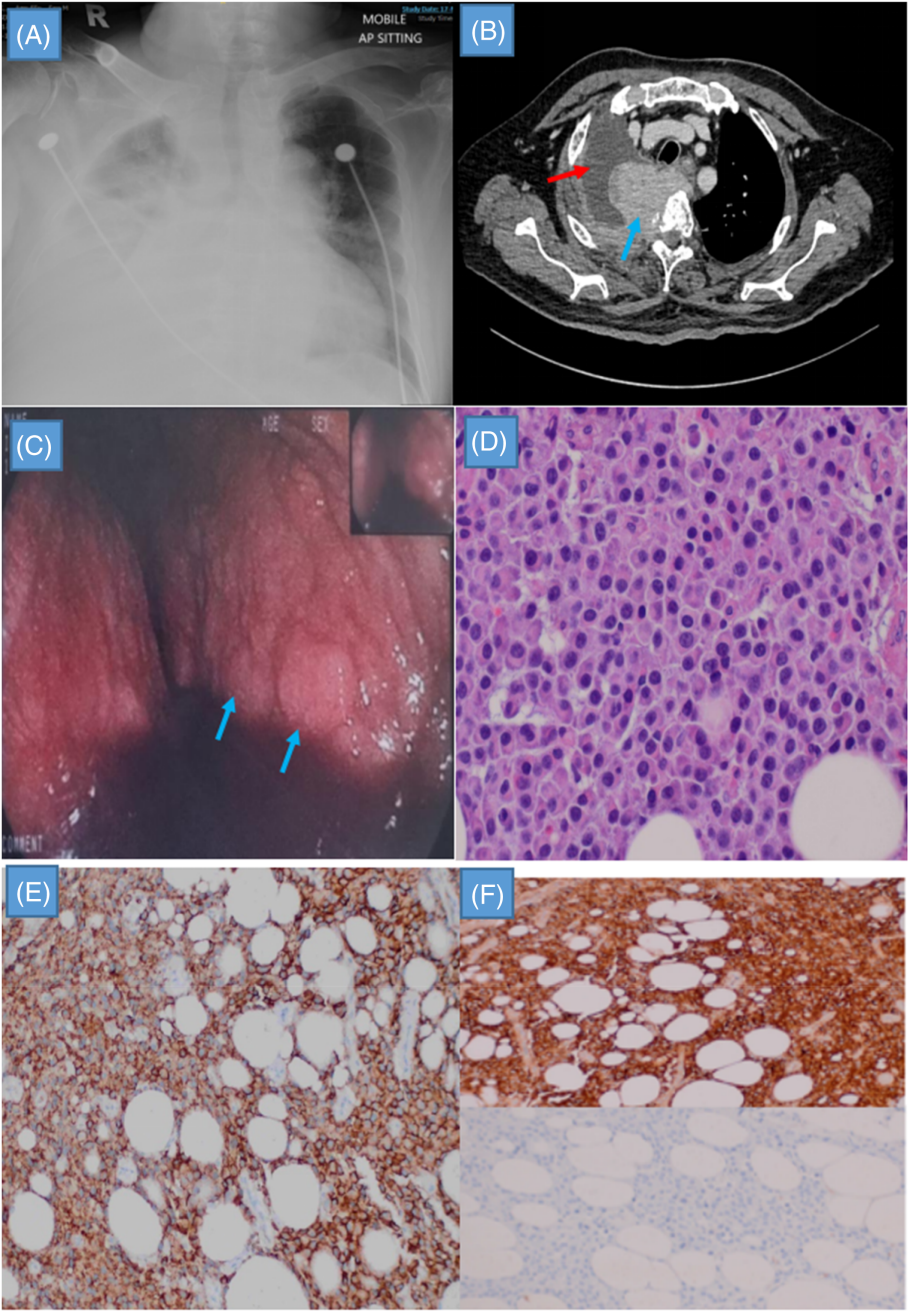
(A) Chest radiograph showing a right pleural effusion with a mass at right upper zone. (B) Computed tomography (CT) of the thorax demonstrating a right upper zone mass with intrapleural extension (blue arrow) and surrounding right pleural effusion (red arrow). (C) Medical thoracoscopy (MT) image showing thickened parietal pleura with diffuse irregular nodules (blue arrows). (D) Haematoxylin and eosin stain showing sheets of medium‐sized neoplastic cells with plasmacytoid appearance. (E) The neoplastic cells are strongly positive for CD138. (F) The neoplastic cells show Lambda light chain restriction.

A 65‐year‐old gentleman with hypertension presented with a 1‐month history of non‐productive cough and shortness of breath as well as weight loss of 13 kg for the past 1‐year. He reported no family history of malignancies. Physical examination showed reduced breath sounds and stony dullness on percussion of the right hemithorax. Chest radiograph revealed a right pleural effusion (Figure 1A). Full blood count showed a reduced haemoglobin level at 8.6 g/L, and leucocytosis at 19.2 × 10^9^/L. His blood urea and creatinine levels were raised at 11.1 mmol/L and 152 mmol/L respectively but calcium and albumin/globulin ratio were within normal limits.

He was initially commenced on intravenous antibiotics for presumed community acquired pneumonia with parapneumonic effusion. Diagnostic thoracentesis confirmed an exudative effusion but pleural fluid for acid‐fast bacilli and cultures for bacterial, fungal and mycobacterium tuberculosis were negative. Cytology of pleural fluid show mesothelial cells, neutrophils and lymphocytes without any atypical cells. Pleural fluid adenosine deaminase (ADA) was raised at 69 IU/L. Computed tomography (CT) of thorax revealed a right upper zone pleural mass measuring 4.7 cm × 6.6 cm × 4.1 cm with intraspinal extension and right pleural effusion (Figure 1B). There were multiple lytic bone lesions suggestive of bone metastasis.

Right medical thoracoscopy (MT) revealed areas of thickened parietal pleura with diffuse irregular nodules scattered over both parietal and visceral pleura (Figure 1C). Histopathological examination of parietal pleura demonstrated neoplastic cells with plasmacytoid appearance (Figure 1D). These cells were strongly positive for CD138 (Figure 1E) and showed Lambda light chain restriction (Figure 1F). Besides, serum free light chain essay showed markedly raised lambda free light chain at 19200 mg/L and serum protein electrophoresis of 1.1 g/L at gamma region. A diagnosis of IgG Lambda MM was made and he was promptly commenced on bortezomib, thalidomide and dexamethasone (VTD regime). Follow up CT after 8 months of therapy showed significant reduction in size of pleural mass with resolution of effusion. He remained well to date when he was last reviewed at the outpatient clinic, 1 year after the initial diagnosis of MM with no recurrence of pleural effusion.

### Case 2

**FIGURE 2:**
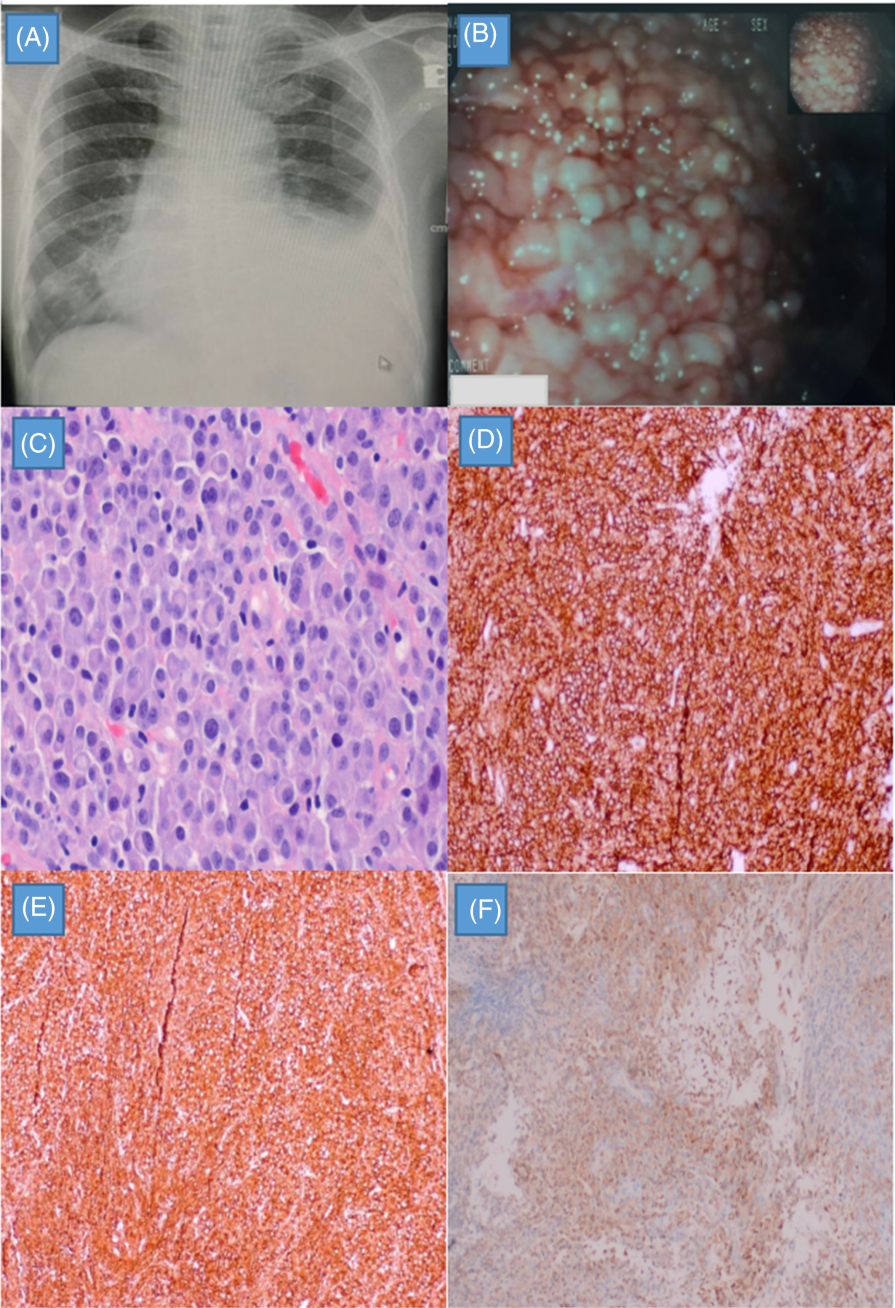
(A) Chest radiograph showing a left sided pleural effusion. (B) Diffuse nodular masses seen along the parietal pleura during medical thoracoscopy (MT). (C) Haematoxylin and eosin stain of pleural biopsy revealing sheets of medium‐sized neoplastic cells with plasmacytoid appearance. **(**D) The neoplastic cells are strongly positive for CD138. (E, F) The neoplastic cells show Lambda light chain restriction.

A 75‐year‐old gentleman with hypertension and IgA Kappa MM diagnosed 4 years ago presented with new‐onset left pleural effusion. He complained of non‐productive cough with left sided pleuritic chest pain and breathlessness for the past 3 weeks. He lost 4 kg for the past month, attributed to poor appetite. His symptoms persisted despite courses of oral antibiotics. Physical examination revealed reduced breath sound and dullness on percussion of the left hemithorax. Chest radiograph confirmed a left pleural effusion (Figure 2A). Thoracentesis showed an exudative effusion but like the previous case, all other investigations including cultures and cytology were negative. Cytospinned pleural fluid samples showed mainly reactive mesothelial cells with a few singly dispersed atypical cells which displayed enlarged hyperchromatic nuclei and scanty cytoplasm. Cell blocks showed mainly mesothelial cells and a few plasmacytoid cell. He is an active smoker of 20‐pack‐years. Further history revealed that he initially received 6 cycles of cyclophosphamide, thalidomide, and dexamethasone (CTD regime) for MM 4 years ago but relapsed a year later where he was given another 2 cycles of bortezomib, thalidomide, and dexamethasone (VTD regime) followed by 6 cycles of bortezomib, lenalidomide and dexamethasone (VRD regime). He was then commenced on maintenance lenalidomide and dexamethasone.

MT revealed multiple discrete nodular masses along the parietal pleura and diaphragm (Figure 2B), where biopsies were taken. Histopathological examination revealed neoplastic cells with plasmacytoid appearances (Figure 2C). The cells stained strongly positive for CD138 (Figure 2D,E) and showed Lambda light chain restriction (Figure 2F), consistent with MM. With the diagnosis in mind, he was referred to the haematology team. Daratumumab, bortezomib, and dexamethasone (DVD regime) was given, which was accompanied by clinical improvements together with resolution of pleural effusion on repeated chest radiographs. He was in remission throughout his follow up period for 18 months but unfortunately defaulted his clinic appointments for the past year.

## DISCUSSION

As mentioned, true myelomatous pleural effusion is a rare clinical entity. Direct pleural involvement by myeloma cells, myelomatous thoracic duct infiltration, and/or invasion of plasmacytomas from chest wall or adjacent skeletal lesions are postulated mechanisms for myelomatous pleural effusion.^3^ Previous studies suggested IgA MM as the most common subtype of myelomatous pleural effusion (up to 80% of cases) although newer reports described IgG myelomatous pleural effusion as well.^5^, ^6^, ^7^ Our first and second patient had IgG and IgA MM respectively. The subtype of myelomatous pleural effusion might confer prognostic implications although studies are needed to prove this hypothesis. Nevertheless, the presence of a myelomatous pleural effusion is generally associated with a poor prognosis, with literature reporting a median survival of lesser than 4 months.^8^

A confident diagnosis of myelomatous pleural effusion can be made through: (a) presence of atypical plasma cells in pleural fluid samples, (b) presence of monoclonal protein on pleural fluid electrophoresis, and (c) histological confirmation using pleural biopsy specimens or autopsy.^7^ However, pleural fluid cytology has a diagnostic sensitivity of only about 60% in all malignancies.^9^ Different techniques have been used to enhance the diagnostic yield of pleural fluid cytology, from using pleural fluid electrophoresis to immunocytochemistry and flow cytometry.^10^ These methods are not available in all centres, especially in resource‐limited settings. The idea of acquiring larger pleural samples with preserved tissue architecture has promoted the idea of performing MT for MM patients with pleural effusion, especially when initial investigations were not diagnostic. Both our cases had negative cytological examinations and were only successfully diagnosed when MT were utilized to provide pleural biopsy samples for histopathological analyses.

Elevated pleural fluid ADA level (with various cut‐off levels but generally above 30 U/L) is commonly used as an adjunct test for the diagnosis of tuberculous pleural effusion (TBE) in endemic regions.^11^ Nevertheless, raised ADA levels have been reported in MM patients with pleural effusion as well.^12^ Postulated mechanisms include secondary pleural infection and inflammation leading to recruitment and maturation of T lymphocytes and consequent raised ADA levels. Hence, elevated pleural fluid ADA levels should not deter healthcare providers from offering other standard‐of‐care tests such as MT, especially when other clinical parameters are not consistent with TBE. In our first case, despite a raised pleural fluid ADA level, we rightly opted to perform MT as our patient had no overt risk factors for tuberculosis. On the other hand, treatment of myelomatous pleural effusion is not well defined but generally include systemic chemotherapy, radiotherapy for local pleural invasion, and/or stem cell transfusion.^1^ Intrapleural chemotherapy using agents such as bortezomib have been described in case reports but has yet to be incorporated into major guidelines.^13^ Fluid control measures such a talc pleurodesis may be necessary in the setting of recurrent fluid re‐accumulation despite MM specific therapy.

In summary, we reported two cases of myelomatous pleural effusion diagnosed by MT. Although rare, MM should be considered in patients with unexplained pleural effusions. MT appears to be a feasible, safe, and minimally invasive tool for diagnosis of myelomatous pleural effusion when other initial investigations are not conclusive.

## AUTHOR CONTRIBUTIONS

Khai Lip Ng, Nai‐Chien Huan Sin Nee Tan contributed to the design and implemenation of the case reports. Khai Lip Ng, Sin Nee Tan, Nai‐Chien Huan wrote the manuscript. Khai Lip Ng, Sin Nee Tan and Mona Zaria Nasaruddin carried out the prodecures mentioned. Noriah Othman interpreted the pathology slides. Jamalul Azizi Abdul Rahaman supervised the project. All authors discussed the study and contributed to the final manuscript.

## CONFLICT OF INTEREST STATEMENT

None declared.

## ETHICS STATEMENT

The authors declare that appropriate written informed consent was obtained for the publication of this manuscript and accompanying images.

## Data Availability

The data that support the findings of this study are available from the corresponding author upon reasonable request.
